# Thirty-Year Lessons from the Newborn Screening for Congenital Adrenal Hyperplasia (CAH) in Japan

**DOI:** 10.3390/ijns7030036

**Published:** 2021-06-29

**Authors:** Atsumi Tsuji-Hosokawa, Kenichi Kashimada

**Affiliations:** 1 Department of Systems BioMedicine, National Research Institute for Child Health and Development, Tokyo 157-8535, Japan; tsuji-a@ncchd.go.jp; 2 Department of Pediatrics and Developmental Biology, Tokyo Medical and Dental University (TMDU), Tokyo 113-8510, Japan

**Keywords:** congenital adrenal hyperplasia, 21-hydroxylase deficiency, newborn screening

## Abstract

Congenital adrenal hyperplasia (CAH) is an inherited disorder caused by the absence or severely impaired activity of steroidogenic enzymes involved in cortisol biosynthesis. More than 90% of cases result from 21-hydroxylase deficiency (21OHD). To prevent life-threatening adrenal crisis and to help perform appropriate sex assignments for affected female patients, newborn screening (NBS) programs for the classical form of CAH have been introduced in numerous countries. In Japan, the NBS for CAH was introduced in 1989, following the screenings for phenylketonuria and congenital hypothyroidism. In this review, we aim to summarize the experience of the past 30 years of the NBS for CAH in Japan, composed of four parts, 1: screening system in Japan, 2: the clinical outcomes for the patients with CAH, 3: various factors that would impact the NBS system, including timeline, false positive, and LC-MS/MS, 4: Database composition and improvement of the screening program.

## 1. Introduction

**Figure 1:**
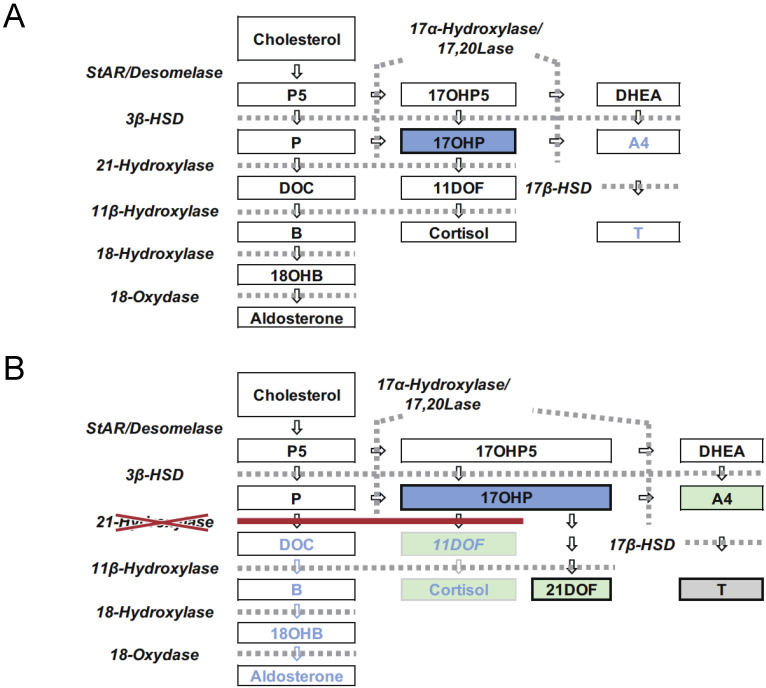
Steroid synthesis in the adrenal cortex (**A**) and the pathophysiology of 21OHD (**B**). P5: Pregnenolone, 17αOHP5: 17-hydroxypregnenolone, P: Progesterone, 17αOHP: 17-hydroxyprogesterone, DOC: Deoxycorticosterone, 11DOF: 11-deoxycortisol, B: Corticosterone, 18OHB: 18-Hydroxycorticosterone, DHEA: Dehydroepiandrostendione, A4: Androstenedione, T: Testosterone. 17αOHP and other green steroids are included in the panel of LC-MS/MS screening in Japan. Steroids written in blue suggest its synthesis is reduced.

Congenital adrenal hyperplasia (CAH) is an inherited disorder caused by the loss or severely impaired activity of steroidogenic enzymes involved in cortisol biosynthesis (Figure 1A,B) [1,2]. More than 90 percent of cases result from 21-hydroxylase deficiency (21OHD) caused by mutations in *CYP21A2*. The prevalence of 21OHD is estimated to be 1:15,000–16,000 in the USA and Europe [3] and slightly lower in Japan (1:18,000) [4,5,6]. The clinical spectrum of the disease ranges from the most severe to mild forms, depending upon the degree of enzyme deficiency [2].

The disease is mainly classified into two forms: classical and nonclassical. The classical form is associated with two major problems: life-threatening adrenal crisis in both sexes and virilization of the external genitalia in 46,XX patients. The classical form is further subdivided into two subtypes, the severest, salt wasting (SW) form, and simple virilizing (SV) form. The SW form is associated with cortisol and aldosterone deficiencies, in which neonates are likely to develop life-threatening adrenal crises with severe hyponatremia and hyperkalemia. Virilization of the external genitalia in newborn females and precocious puberty due to overproduction of androgens by the adrenal cortex are the other major clinical manifestations of the SW and SV forms. However, the clinical phenotypes of the SW type and the SV forms may overlap, and attempts to differentiate them based on endocrinological evaluation without genetic analysis are sometimes inconclusive [2].

To prevent a life-threatening adrenal crisis and help perform appropriate sex assignments for affected female patients, newborn screening (NBS) programs for the classical form of CAH have been introduced in numerous countries [7]. In Japan, the NBS for CAH was introduced in 1989, following that for PKU and congenital hypothyroidism [4].

In contrast to the classical form, the nonclassical form has a milder phenotype in which clinical problems are not obvious during the neonatal period or childhood, generally developing during adolescence or adulthood [1,2]. The prevalence of nonclassical form in Japan is estimated much lower than that in western countries [8,9,10]. Although some of them are screened by the NBS, the screening program is not designed to detect all the newborns with the nonclassical form.

The aim of this review is to summarize the experience of the past 30 years of the NBS for CAH in Japan, comprising four parts: 1, screening system in Japan; 2, clinical outcomes for patients with CAH; 3, factors that would impact the NBS system, including timeline, false positive, and LC-MS/MS; and 4, database composition and improvement of the screening program.

## 2. Screening System in Japan

The NBS in Japan was introduced individually into the prefectural administration according to a government notification by the Ministry of Health and Welfare in 1977. The basis of the NBS system, such as the timeline, and the screening panel are identical in all local governments. After informed consent is obtained from a legal guardian, blood samples are collected by a heel prick blotted on a filter paper from neonates at 4–7 days from birth, and the filter paper samples are immediately sent to a laboratory allocated by the prefectural government.

**Figure 2:**
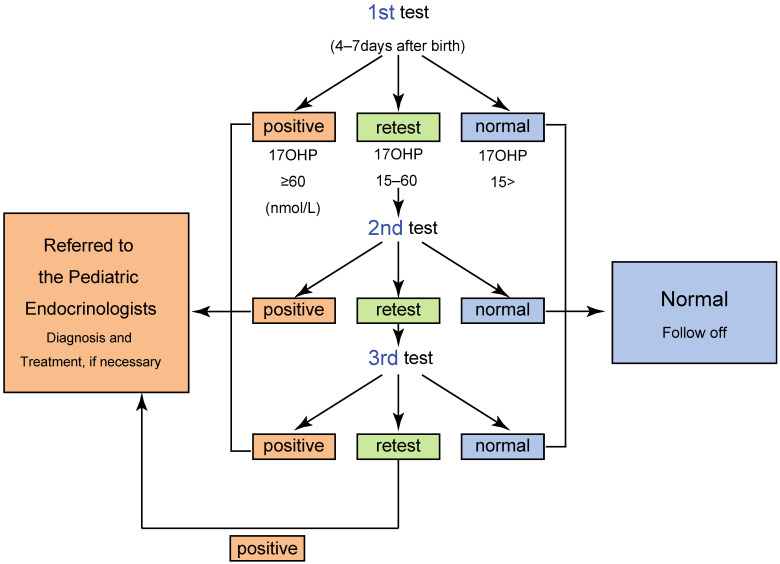
Algorithm of CAH screening in Tokyo.

The details of the screening system are different among laboratories, and as a representative, the screening algorithm in Tokyo was shown in Figure 2. In Tokyo, the 1st screening is divided into two procedures. The level of 17-hydroxyprogesterone (17αOHP) is initially determined by enzyme-linked immunosorbent assay (ELISA) without steroid extraction. We select blood samples in the 97th percentile or higher for 17αOHP values for subjecting the second-tier test, which is carried out after steroid extraction [4,5,11,12]. The cutoff criteria for the second-tier test are shown in Table 1.

The NBS has two different cutoff values: for “screening positive” and for “retest”. When the 17αOHP level is higher than the screening positive cutoff value, the neonate is directly referred to a pediatric endocrinologist for further endocrinological evaluation. Neonates with 17αOHP levels more than the retest cutoff value are retested. When the 17αOHP levels are higher than the retest cutoff value two–three times, the screening is considered positive (Figure 2) [4,5,11,12,13].

In some female patients, blood sampling for the screening is performed ahead of schedule due to atypical genitalia, which is one of the major clinical symptoms in female neonates with 21OHD and is frequently recognized at birth.

To reduce the number of false-positive results in preterm newborns, one of the most serious issues in the screening for 21OHD, some laboratories, including that of Tokyo, employ cutoff values based on gestational age and/or birth weight. The cutoff values were determined according to a pilot study of serum 17αOHP levels in full-term and preterm infants. As a representative screening system in Japan, the algorithm and criteria for the screening in Tokyo are shown in Table 1 and Figure 2 [5], respectively.

For the quality control of the screening, most screening laboratories perform follow-up surveys of the patients who were referred to hospitals. In the surveys, clinical information of the patients, including confirmed diagnosis, is collected from the pediatric endocrinologists at the hospitals [13].

## 3. Clinical Outcomes of the Newborn Screening for CAH in Japan

### 3.1. The Effects of the Screening

The clinical profiles of 21OHD before the introduction of the screening differ remarkably from the current profiles [14]. Before the introduction of the screening, Suwa S et al. conducted a nationwide survey and reported the clinical profiles of 21OHD in Japan. According to the survey, the estimated prevalence of 21OHD was 1/43,764, and the average age in days when the patients firstly visited the hospitals was 1102. In the SW type, the average age of the first hospital visit was 55 days (male: 63 days (range, 1 days to 3 years), female: 47 days (range: 0 days to 3.9 years)), and in the SV form, the average age at first visit was 6.4 years (male: 5.9 years (range: 14 days to 34 years), female: 6.5 years (range: 0 days to 44 years)) (Table 2) [15]. The ratio of male to female was 1:1.5, and the number of male patients was significantly lower than that of females, implying that a substantial number of male patients were missed, i.e., the SV form remained undiagnosed or the fatal cases with the SW form in the neonatal-infantile period. Consistently, the survey revealed that the mortality rate was 10.6% in neonates with the SW form, which is consistent with the reports from other countries [15]. In 46,XX cases, 12.9% were firstly assigned as male because of atypical genitalia and corrected to female sex after the diagnosis of 21OHD [15].

After the introduction of the screening, the clinical outcomes of 21OHD during the neonatal/infantile period were remarkably improved. The average ages at the first visit were 8.2 and 7.6 days (male: 9.2 days, female: 6.0 days) in Sapporo and Tokyo, respectively (Table 2) [6,13]. To date, no fatal cases have been identified.

Although the follow-up surveys and the screening systems are not designed for detecting false-negative cases, based on a survey for the literature and the annual reports from NBS programs, no false-negative cases have been reported since the introduction of the screening [5,6,13]. We presume that the sex of all 46,XX cases was correctly assigned.

### 3.2. The Progression of Salt Wasting during the First Two Weeks of Life

Adrenal crisis is a life-threatening medical emergency, and eradicating the lethal cases of 21OHD is one of the major goals of newborn screening [16]. Although the fact that there were no reported fatal cases suggests the primary goal of the screening has been accomplished, 37.4% of 21OHD neonates already developed severe salt wasting, which is defined by Na < 130 mEq/L, K > 7 mEq/L, on arrival at medical hospitals in Tokyo screening [13]. Furthermore, some of the 21OHD neonates exhibited life-threatening salt wasting, such as more than 10 mEq/L of serum K [13].

Severe adrenal crisis during the neonatal to early infantile period would cause neurological comorbidities. According to the nationwide survey before the introduction of NBS in Japan, a substantial number of the 21OHD patients revealed to have neurological comorbidities including intellectual disability and epilepsy. The prevalence associated with the SW form was higher, 18.5%, than with the SV form, 9.4%, suggesting that delayed diagnosis of adrenal crisis causes intellectual disability [17]. Consistently, in the retrospective study from the U.K., where NBS for 21OHD is not introduced, more than 20% of the SW-type 21OHD patients developed learning difficulties [14]. Those suggest that just eradication of lethal cases would not be sufficient for the goal of the 21OHD screening, and avoiding severe adrenal crisis should be considered.

**Figure 3:**
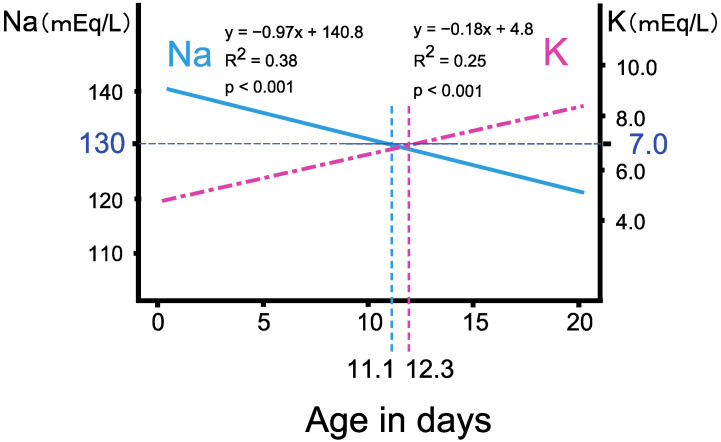
Clinical features of serum sodium (Na) and potassium (K) levels in 21OHD neonates. Retrospective analysis of the NBS in Tokyo revealed that, in classical 21OHD patients, the serum Na and K levels linearly deteriorated with age in days, and the age when the regression lines reached Na < 130 mEq/L, K > 7 mEq/L approximately coincided at 11.1 and 12.3 days, respectively. (Modified from Gau et al., 2020 [13]).

Retrospective analysis of the follow-up survey of the NBS in Tokyo revealed that, in classical 21OHD patients, the serum Na and K levels linearly deteriorated with age in days, and the age when the regression lines reached Na < 130 mEq/L, K > 7 mEq/L approximately coincided at 11.1 and 12.3 days, respectively [13] (Figure 3). The risk of developing severe salt wasting increases during the second week of life without a threshold, and, therefore, an early intervention, ideally during the first week of life, is desirable [13,18,19].

### 3.3. Triage of the Neonates with Salt Wasting by Body Weight Change

**Figure 4:**
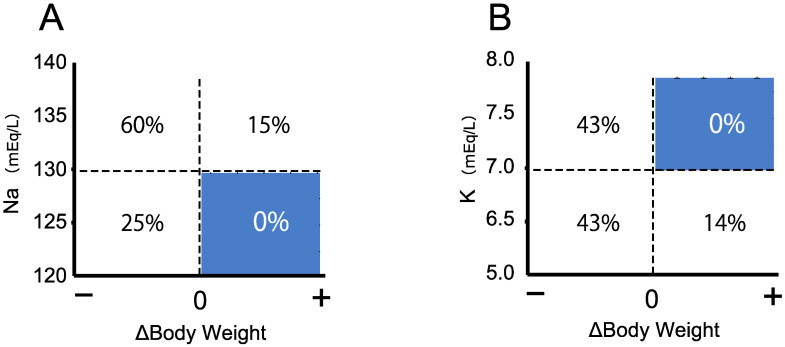
Body weight change from birth is an excellent predictor of 21OHD and the risk of severe salt wasting. Body weight data of 21OHD patients at 7–14 days after birth were collected, and the change in body weight from birth weight was examined. None of the 21OHD patients with severe salt wasting (Na < 130 meq/L or K > 7.0 mEq/L) exhibited increased body weight (**A**,**B**).

The follow-up survey in Tokyo revealed that from the second week of life, changes in body weight provide a useful index in the evaluation of neonates with positive CAH screening results [13]. Neonates with decreasing body weight from the birth weight are likely to have classical 21OHD, and neonates with increasing body weight after birth are more likely to be false positives [13]. Furthermore, even in cases of 21OHD, the possibility of developing severe salt wasting, such as hyponatremia (<130 mEq/L) or hyperkalemia (>7 mEq/L), is extremely low without loss of body weight (Figure 4). Contrary to body weight change, the relevance of predicting severe salt wasting based on the 17αOHP level is extremely low because the 17αOHP level is not associated with Na or K levels [13].

Although the findings of body weight change in patients cannot be the direct criteria for the CAH screening protocol, they may assist in some individual cases, e.g., for triaging a neonate with a positive result who is living in a region with limited access to a pediatric endocrinologist or in which there is no CAH screening.

## 4. Potential Issues of Testing Practices in the Newborn Screening for CAH in Japan

### 4.1. The Timeline of the Newborn Screening for 21OHD

The timeline of the NBSs is becoming earlier worldwide because newly added inborn errors to the screening panel require early intervention immediately after birth. In the U.S., SIMD (Society for Inherited Metabolic Disorders) defines the critical condition as a condition in which serious symptoms may present acutely in the first weeks of life with a short pre-symptomatic window and require immediate treatment to mitigate morbidity and mortality [20]. More than 10 inborn errors of organic acid disorders and fatty acid oxidation disorders are involved in the list, and the SIMD recommends considering the list as an important starting point for discussion between clinicians and laboratories [20]. Accordingly, blood samples for screening are collected 48 h after birth in the U.S., and the recommended age in days when the first results are obtained should be seven [21]. Indeed, in 2018, 64% of the first results were available within 5 days after birth (Table 3) [22]. The situation is similar in the EU, and, in most countries, blood sampling starts 72 h after birth (Table 3) [23].

In the NBS for 21OHD, several factors should be considered in terms of timing for the blood sampling. Especially, given the rate of 37.4% neonates with severe salt wasting in Japan, earlier sampling can be discussed for the prevention of life-threatening salt wasting. However, an increase in serum 17αOHP level has been observed in unaffected neonates during the first 1–2 days of life, and there is evidence of false negatives associated with the early collection of specimens in the U.S. [24]. Further, the timeline is determined by various factors of other diseases in the screening panels, which are different among countries (Table 3). For optimizing the timeline of the screening, we need careful discussion continuously.

### 4.2. High Rate of False Positive

For the 17αOHP measurement, immunoassays have been used because of their sensitivity, cost, and simplicity. However, immunoassays lead to high rates of false positives, seriously affecting the screening efficiency [5,18,26].

**Figure 5:**
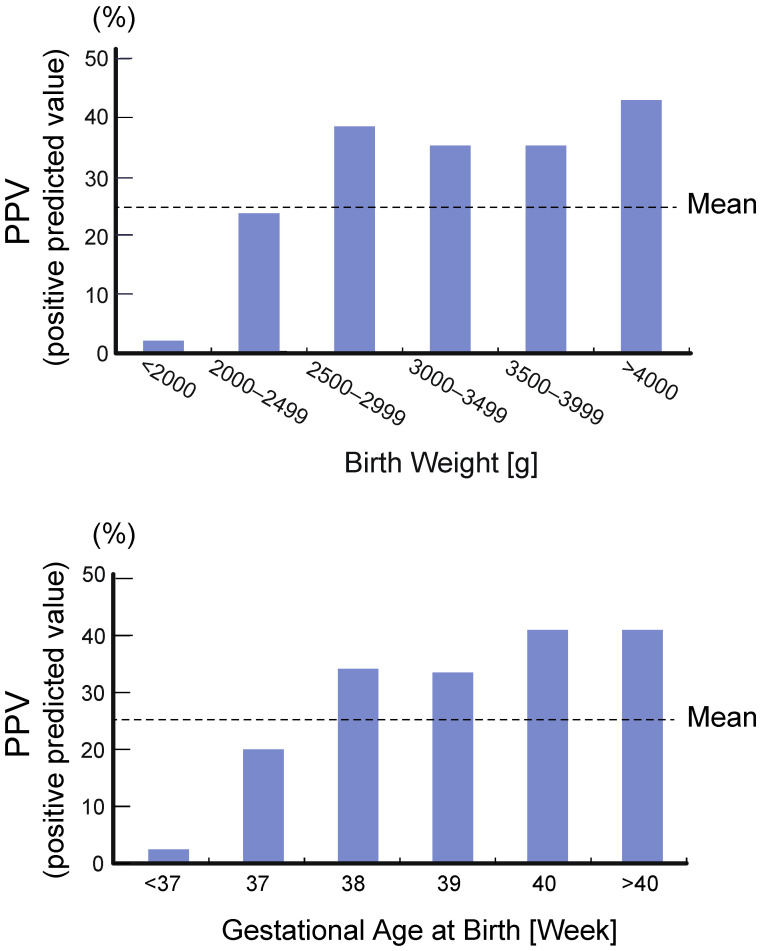
PPV (positive predicted value) of CAH screening in Tokyo according to the birth weights and the gestational ages of newborns judged as positive. (Modified from Tsuji et al., 2015 [5]).

One of the major reasons is the cross-reactivity with steroids, such as 17-hydroxypregnenolone sulfate and 15β-hydroxylated compounds, which is high in preterm infants, and the ratio of false positives is extremely high in preterm infants. To minimize false positives, cutoff points stratified by gestational age and/or birth weight have been used in some screening systems. Although the stratified cutoff improves positive predictive value (PPV) to some extent, its efficiency is limited [5,27,28,29,30]. In the Tokyo system, gestational age and birth weight cutoff points have been used since the introduction of the NBS (Table 1). While the average PPV in Japan was reported as 6.6%, the Tokyo screening program achieved 25.8% (Figure 5). On the other hand, the PPV in preterm infants with a gestational age of ≤37 weeks was only 2% [5].

Another cause for the high false-positive rate is the nature of 17αOHP itself. Historically, 17αOHP was originally considered as the pathogenic androgen in cases of 21OHD rather than as a diagnostic marker, and it has several shortcomings as a diagnostic for 21OHD [31]. The level of 17αOHP is high in cord blood during the first 1–2 days of life, and stress from other illnesses may result in the 17αOHP remaining high in unaffected neonates. Furthermore, in other forms of CAH, including 11-hydroxylase deficiency (11OHD), 3β-hydroxysteroid dehydrogenase deficiency (3βHSDD), and P450 oxidoreductase deficiency (PORD), 17αOHP may be elevated to almost the same level as that of 21OHD [32]. For further improving PPV in 21OHD screening, measuring other biomarkers with high specificity for 21OHD would be required.

### 4.3. LC-MS/MS Analysis of 17αOHP as a Second-Tier Test and Diagnostic Test for 21OHD

To improve PPV, an alternative methodology should measure disease-specific markers other than 17αOHP or has high specificity for the target steroids. When used appropriately under highly regulated conditions, liquid chromatography-tandem mass spectrometry (LC-MS/MS) is considered as the gold standard for steroids assays [33,34,35,36,37,38,39], and the guideline of the Endocrine Society have recommended to employ LC-MS/MS for measuring 17αOHP of the second tier in neonatal screening since 2018 [16].

In addition to its specificity, the advantage of LC-MS/MS is the capability for the simultaneous assay of multiple steroids [33,34,36]. In Japan, a steroid profile panel from Siemens Healthineers AG (Frankfurt, Germany), “MS^2^-screening CAH” was developed for the CAH screening. Five steroids were selected for the panel: 17αOHP, 21-deoxycortisol (21-DOF), 11-deoxycortisol (11-DOF), 4-androstenedione (4AD), and cortisol (F). Accordingly, the cutoff criteria of the LC-MS/MS assay are not solely based on 17αOHP, but on 21DOF and the ratios of steroids, such as (17αOHP + 4AD)/F, 11DOF/17αOHP (Table 4) [33]. The combination of highly specific LC-MS/MS and simultaneous assays of five steroids is expected to dramatically improve the efficiency of the screening [33,34,36].

Indeed, the outcomes of LC-MS/MS are excellent. In 2018, the LC-MS/MS assay for 21OHD was employed in 5 of 37 prefectural laboratories in Japan. In immunoassay screening, out of 653 subjects with positive results, there were 38 confirmed cases of 21OHD, resulting in a PPV of 5.8% (38/653). On the other hand, the PPV in LC-MS/MS screening was 40.0% (6/15), indicating that the specificity of LC-MS/MS is remarkable [40]. Accordingly, in 2018, the Ministry of Health, Labor, and Welfare in Japan added LC-MS/MS to the list of recommended methodologies for the second-tier test of 21OHD screening [41].

In addition to improving the efficiency of 21OHD screening, the steroid profile assay by LC-MS/MS may bring further advantage to the screening, that is, assisting definitive diagnosis of 21OHD [34]. Although 21OHD can be diagnosed endocrinologically, the procedures and cutoff criteria are complicated because other rare forms of CAH, such as 11OHD, PORD, and 3βHSDD, should be differentiated from the diagnosis of 21OHD as we described in the previous section [32,42,43,44,45,46,47,48]. The nonspecific increase in 17αOHP levels in other forms of CAH has been considered as a potential clinical pitfall.

Currently, reliable methods for differentiating 21OHD from other forms of CAH are an adrenocorticotropic hormone (ACTH) stimulation test [16], urine steroid profile analyses using gas chromatography mass spectrometry [49], and genetic test, which cannot be used as a first-line diagnostic test because the procedure of *CYP21A2* gene analysis is extremely complicated [50,51,52].

It has been suggested that the ratios of steroids (17αOHP + 4AD)/F, 11-DOF/17αOHP, and 21-DOF may be more specific biomarkers for the diagnosis of 21OHD than that of 17αOHP and are expected to differentiate 21OHD from other types of CAH in which 17αOHP levels are elevated, such as 3βHSDD, 11OHD, and PORD. Although there are few reports of the levels of (17αOHP + 4AD)/F, 11-DOF/17αOHP, or 21-DOF in these forms of CAH, some cases suggest the potential usefulness of the five steroids in the screening panel. 21DOF would not be elevated in 3βHSDD because, in a model of partial 3βHSD deficiency preterm infants, 21DOF is not grossly elevated [34]. In patients with 11-OHD, 21-DOF levels are reported to be normal, and 11-DOF is markedly elevated, presumably increasing the 11-DOF/17αOHP ratio [53]. Urinary steroid profile analyses of PORD suggested that the ratio of pregnanetriolone (Ptl)/tetrahydrocortisone steroids (THEs) and a specific cutoff of 11β-hydroxyandrosterone (11HA) would be useful for differentiating PORD from 21OHD. Ptl, THEs, and 11HA are metabolites of 21DOF, 11DOF + cortisol, and 4AD, respectively, which are included in the LC-MS/MS screening panel [54].

Accordingly, in combination with other clinical symptoms and signs, such as poor body weight gain and high ACTH, 21OHD can be diagnosed based on the results of NBS by LC-MS/MS [31,34]. However, we cannot directly apply the screening criteria to the diagnostic criteria, and for establishing the diagnostic criteria, an accumulation of the cases is required.

## 5. Database Composition and Improvement of Screening Program

For better and more efficient management of the CAH, the screening programs need persistent improvement in quality. By examining reliable follow-up studies, the outcomes and the experiences of the screening should be retrospectively evaluated and shared among the screening laboratories [25,55,56]. For short-term outcomes, most laboratories and local governments have introduced follow-up surveys in Japan, using the results for more efficient screening by decreasing false positives and early availability of screening results.

On the other hand, the assessment of long-term outcomes for CAH patients identified by screening is challenging. A nationwide registry system is required to establish efficient long-term follow-up systems. In Japan, the current screening system depends on each local government; thus, the demanding task of organizing a cross-regional collaborative system that involves local governments, local laboratories, and medical institutes is required.

Recent studies have revealed that 21OHD patients have substantial risks for metabolic syndrome in adulthood [57,58,59,60]. The metabolic syndrome in 21OHD has been assumed to be due to long-term glucocorticoid therapy [61]. However, other causes, such as fetal environments, have also been suggested [62,63], and the pathophysiology of the condition remains unknown. Further, the quality of life of 21OHD patients in adulthood is largely unknown. Especially in female patients, their gender issues should be clarified in detail [64,65,66]. As mentioned previously, before the introduction of the screening, 21OHD patients had substantial risks for neurological sequelae, which are presumably caused by the adrenal crisis during the neonatal period [14,17]. Therefore, the introduction of NBS would reduce the risk for neurological sequelae [67], but currently, available data is limited. We should keep in mind that, even after the introduction of the screening, there are a substantial number of 21OHD patients who developed severe salt wasting before the introduction of therapy. Further, a recent study suggested the number of hyponatremic episodes is an independent risk for lower IQ, suggesting that for optimizing the management of 21OHD patients during childhood, preventing episodes of severe adrenal crisis is crucial [68]. Thus, clarifying long-term outcomes will provide valuable information for improving the screening logistics.

Long-term outcomes will also provide valuable insights for evaluating the cost-effectiveness of screening. Although several analyses have been performed economically, they were based on short-term outcomes with various analytical models, leading to inconsistent results [69,70,71]. In Japan, economic analyses based on detailed clinical data have not been performed. To better understand the cost-effectiveness of screening, comprehensive approaches based on long-term outcomes are essential.

Despite not covering 21OHD patients, the introduction of several registry systems for rare congenital diseases has encouraged us. In the EU, some international collaboration-based registry systems for rare congenital diseases, such as the European Registry and Network for Intoxication type Metabolic Diseases (E-IMD) and the European Registry and Network for Homocystinurias and Methylation Defects (E-HOD), have been established [25,72,73,74,75]. Further, the Japanese Society for Inherited Metabolic Disease has successfully introduced the registry system, “JaSMIn”, for patients with inherited metabolic disease (https://www.jasmin-mcbank.com/, visited 23 April 2021). The limitation of this registry system is the unknown coverage rate due to voluntary patient registration. However, the registry is designed to cover a broad spectrum of rare inherited metabolic diseases that can be discovered by NBS, and it will provide valuable insights, enabling feedback on newborn screening in the future, including economic aspects.

Ideally, for establishing feedback systems with long-term follow-up surveys, close collaboration among the screening laboratory professionals, pediatricians, primary care providers, and clinical epidemiologists is essential [55,56,76,77]. Currently, to share the outcomes of the screening and updating of technical information, the Japan Society for Neonatal Screening has a collaborative laboratory integrated committee (Gijutsubu-kai). We expect that with this committee, pediatric endocrinologists and local governments would be able to construct large collaboration-based reports and infrastructure. A nationwide registry system in which all results of infants are registered and evaluated periodically would lead to further methodological improvements.

## Figures and Tables

**Table 1:** Criteria of CAH mass screening in Tokyo.

<Criteria According to the Gestational Age>					
Gestational age at birth (weeks) *^1a^		≤29	30–34	35–36	≥37
Corrected gestational age (weeks) *^1b^		≤31	32–35	36–37	≥38
<Criteria According to Weight> *^2,^*^3^					
Body weight (g)		≤999	1000–1999	2000–2499	≥2500
Cutoff level of 17αOHP [n·mol/L]	Retest *^4^	60	45	24	15
	Positive *^5^		60	60	60

*^1a^ Samples collected before the age of 7 days, *^1b^ Samples collected at the age of 7 days or after, *^2^ 1st test: body weight = birth weight, 2nd test and after: body weight = corrected body weight calculated by the formula as below. Corrected body weight at test (g) = birth weight (g) + (age at test − 7) × 20 (g). *^3^ For infants born small or large for gestational age, either the criteria of gestational age (corrected gestational age) or body weight was applied, whichever was a lower value. Since 2012, criteria according to weight have not been used, and solely gestational age-stratified cutoff has been used. *^4^ recall for the second (or the third) test of the screening. *^5^ refer to hospitals for further endocrinological examinations.

**Table 2:** Age at diagnosis before and after implementation of the screening.

	Before CAH Screening *			After CAH Screening **		
	Male	Female	Total	Male	Female	Total
SW type	63 days (120)	47 days (96)	55 days (216)	9.0 days (55)	6.2 days (45)	7.6 days (100)
SV type	5.0 yrs (39)	6.5 yrs (150)	6.4 yrs (189)			

Numbers in parentheses indicate numbers of the subjects, *, **: according to the data reported by Suwa et al., 1994 [15] and Gau et al., 2020 [13], respectively.

**Table 3:** Summary of newborn screening in European countries, Oceania, and the U.S. (modified table from reference [25]) and the following website (https://www.hrsa.gov/advisory-committees/heritable-disorders/newborn-screening-timeliness.html, https://newbornscreening.hrsa.gov/your-state#w, https://www.newsteps.org/resources/data-visualizations/newborn-screening-status-all-disorders, visited date, “23 April 2021”).

Countries *^1^	Approximate Population (Million)	Screening Panel					Interval Birth-Sampling				Interval Sampling Analysis					
		CAH	CH	PKU	GAL	AAD, OA, FAOD	<48 h	48–72 h	72–96 h	>96 h	1 d	2 d	3 d	4 d	5 d	>6 d
Austria	8.8	x ^*2^	x	x	x	>6	x	x			x	x	x			
Belgium	10.5	x	x	x	x	>6		x	x	x		x				
Denmark	5.6	x	x	x	x	>6		x			x	x				
France	67	x	x	x	x	P *^2^		x				x	x			
Germany	80	x	x	x	x	>6	x	x				x	x			
Netherlands	17.8	x	x	x	x	>6			x		x	x	x			
Spain	46.5	x	x	x	P	>6	x	x	x				x	x	x	x
Sweden	10	x	x	x	x	>6		x			x	x	x			
Switzerland	8.1	x	x	x	x	1–6			x			x				
Finland	5.5		x	x		>6		x *^4^	x	x	x	x	x	x	x	
Greece	10.5		x	x	x			x								x
Hungary	10		x	x	x	>6		x					x	x		
Ireland	4.9		x	x	x				x	x	x	x				
Italy	60.5		x	x	P	P		x			x	x	x	x		
Norway	5.3		x	x		>6		x					x			
Portugal	10.3		x	x		>6		x			x	x	x			
U.K.	66.6		x	x		1–6				x			x	x		
U.S. *^3^	328.2	50/50	50/50	50/50	50/50	50/50	x				x	x				
JPN	126.3	x	x	x	x	>6				x	x	x	x			

*^1^ European countries whose population is approximately more than 5 million were listed. *^2^ x (in screening panel section) = in screening panel, P = pilot/regional screening. *^3^ In the United States section, the number of states that include the disease in the screening panel is listed. In the AAD, OA, and FAOD section, states that implemented more than six of the metabolic disorders were counted. The interval between birth, sampling, and analysis of U.S. is recommended timeline. *^4^ Cord blood is used for some of the screening.

**Table 4:** Cutoff level of 17αOHP and other steroids assayed by LC-MS/MS in Saitama, Sapporo and Tokyo, Japan *^1^.

Screening Positive Cutoff Level					
Prefecture		Saitama	Sapporo	Tokyo	
				Criteria A *^2,^*^3^	Criteria B *^2,^*^3^
17αOHP (ng/mL)	Term birth	>20	>20	>5	>5
	Preterm birth	>30	>50		
21DOF (ng/mL)		>1.0	>2.0	>1.0	
(17αOHP + 4AD)/F					>2.0
11DOF/17αOHP					<0.1
**Retest Cutoff Level**					
Prefecture			Saitama *^2^	Sapporo *^2^	Tokyo *^2^
17αOHP (ng/mL)			>1.0	>2.5	>1.5
(17αOHP + 4AD)/F			>0.1	>0.1	>0.3
11DOF/17αOHP			<0.3	<0.2	<0.3

*^1^ The algorithm of the screening is the same as shown in Figure 1. When the retest values are documented twice, the patients are judged as positive. *^2^ The result is judged as positive or retest when all parameters meet the criteria. *^3^ The patient with the result that meets criteria A or B is considered as screening positive.

## Data Availability

The data presented in this study are available on request from the corresponding author. The data are not publicly available due to privacy policy.

## Abbreviations

17αOHP	17-Hydroxyprogesterone
yrs	Years
CAH	Congenital adrenal hyperplasia
CH	Congenital hypothyroidism
PKU	Phenylketonuria
GAL	Galactosemia
AAD	Disorders of amino acid metabolism
OA	Disorders of organic acid metabolism
FAOD	Disorders of fatty acid metabolism
